# What Is Patent Flour?

**Published:** 1882-04

**Authors:** 


### WHAT IS PATENT FLOUR?

PATENT flour is now coming into general use, and many of our readers may be interested in reading the following explanation of what it is and how it is made, taken from the *Prairie Farmer:*

Until recently, the best flour was made from winter wheat ; or, rather the flour made from winter wheat sold for the most money because it was white. But it consisted for the most part of the starch of the grain, while the most of the gluten (the most nutritious part of the grain) went into the middlings.

In grinding spring wheat, so much bran remained in the flour that it was too dark to suit the taste of consumers. But the middlings, which sold at a low price, has become the most desirable part of the grain.

Middling purifiers—by which the bran is separated from the middlings—have made a revolution in the business of milling.

By the new process the wheat is ground as before, except that the efforts of the miller are directed to obtaining the most middlings possible, and these are placed upon large horizontal sieves which are constantly agitated, while, at the same time, by ingenious devices, a draft of air is rushed up through the sieves which carries off the bran.

The purified middlings are then reground, and the product is "patent" flour, containing the glutenous, or most nutritious portion of the grain. Thus it is explained why the hard spring wheats of Minnesota, Northern Wisconsin and Dakota bring the highest price in the market, whereas only a few years ago they commanded only the lowest price.

